# Supplementary material for: Integrin α2 and β1 Cross-Communication with mTOR/AKT and the CDK-Cyclin Axis in Hepatocellular Carcinoma Cells
Source: Cancers (Basel). 2022 May 14;14(10):2430. doi: 10.3390/cancers14102430 (PMC9139686; doi:10.3390/cancers14102430)

Figure S1

Protein bands, corresponding to figure 7

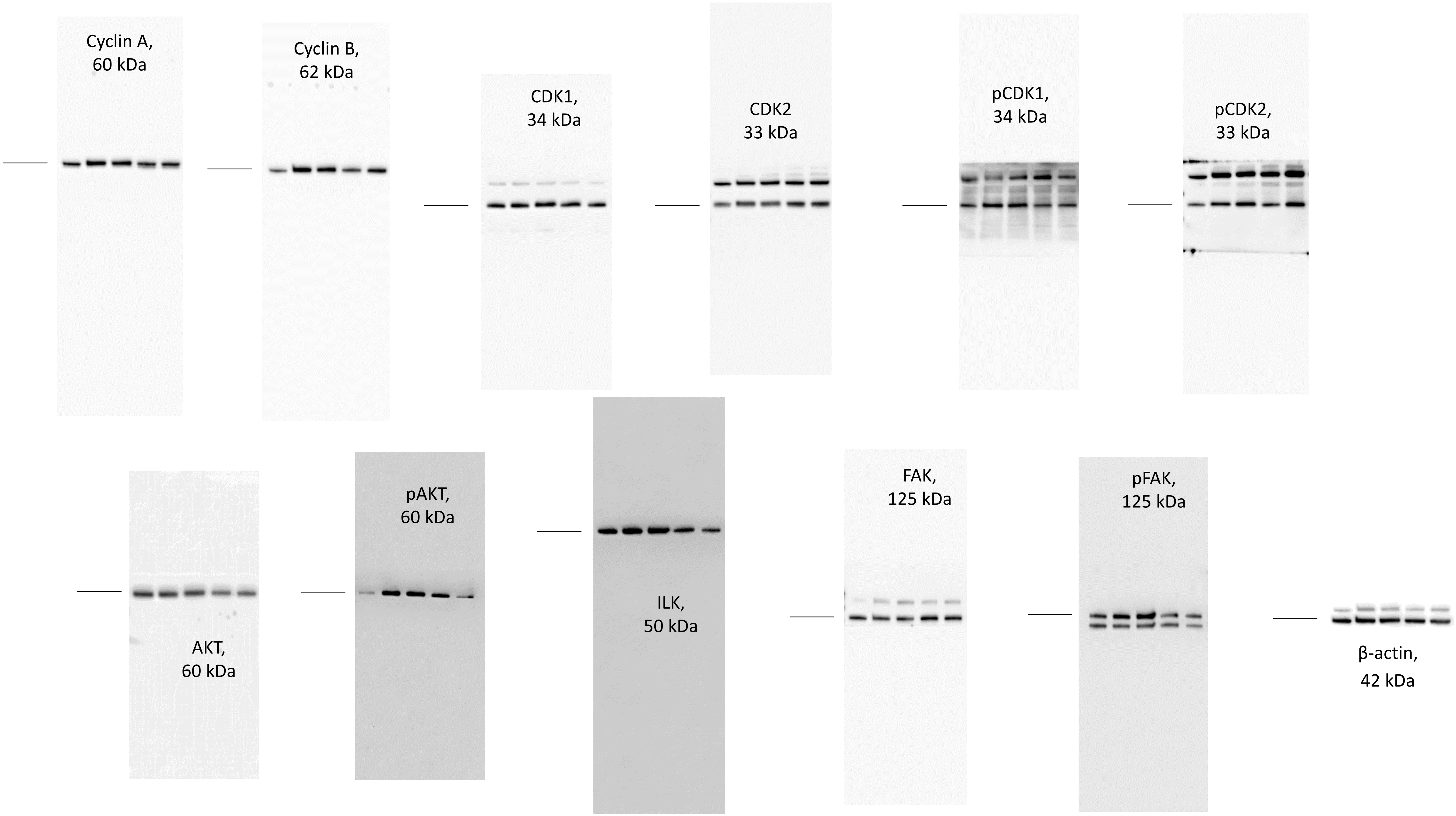

Protein bands, corresponding to figure 7 (continued)

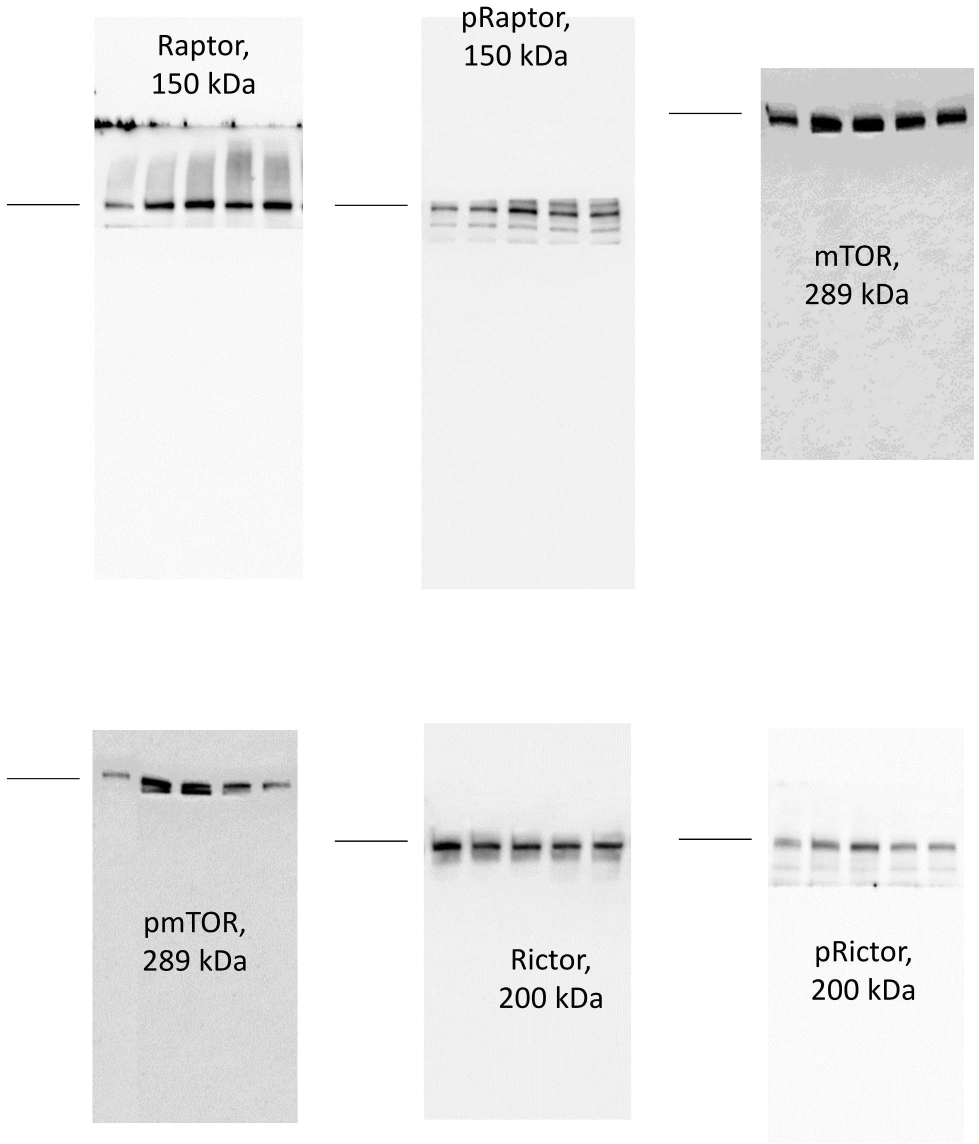

Protein bands, corresponding to figures 4 and 6

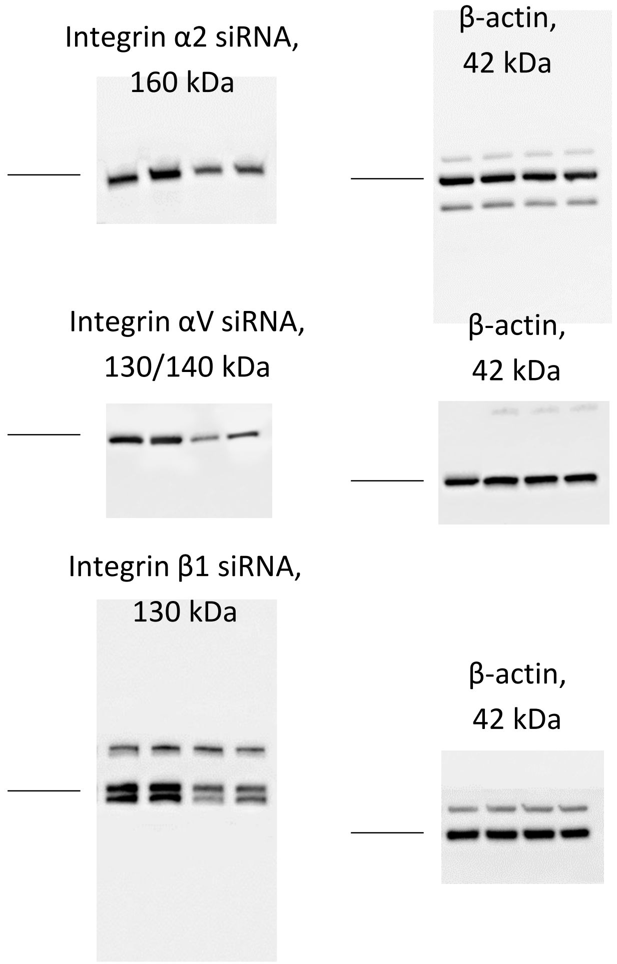

Protein bands, corresponding to figure 8

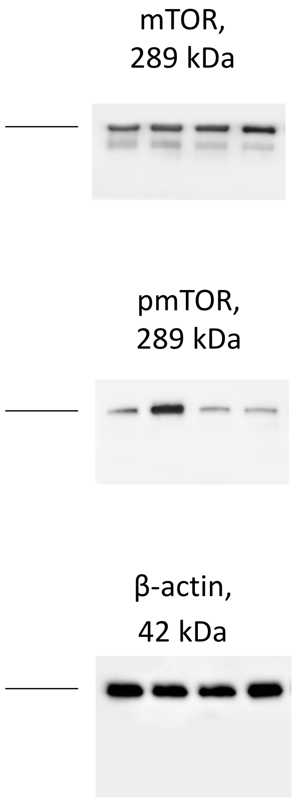

Immunoprecipitation

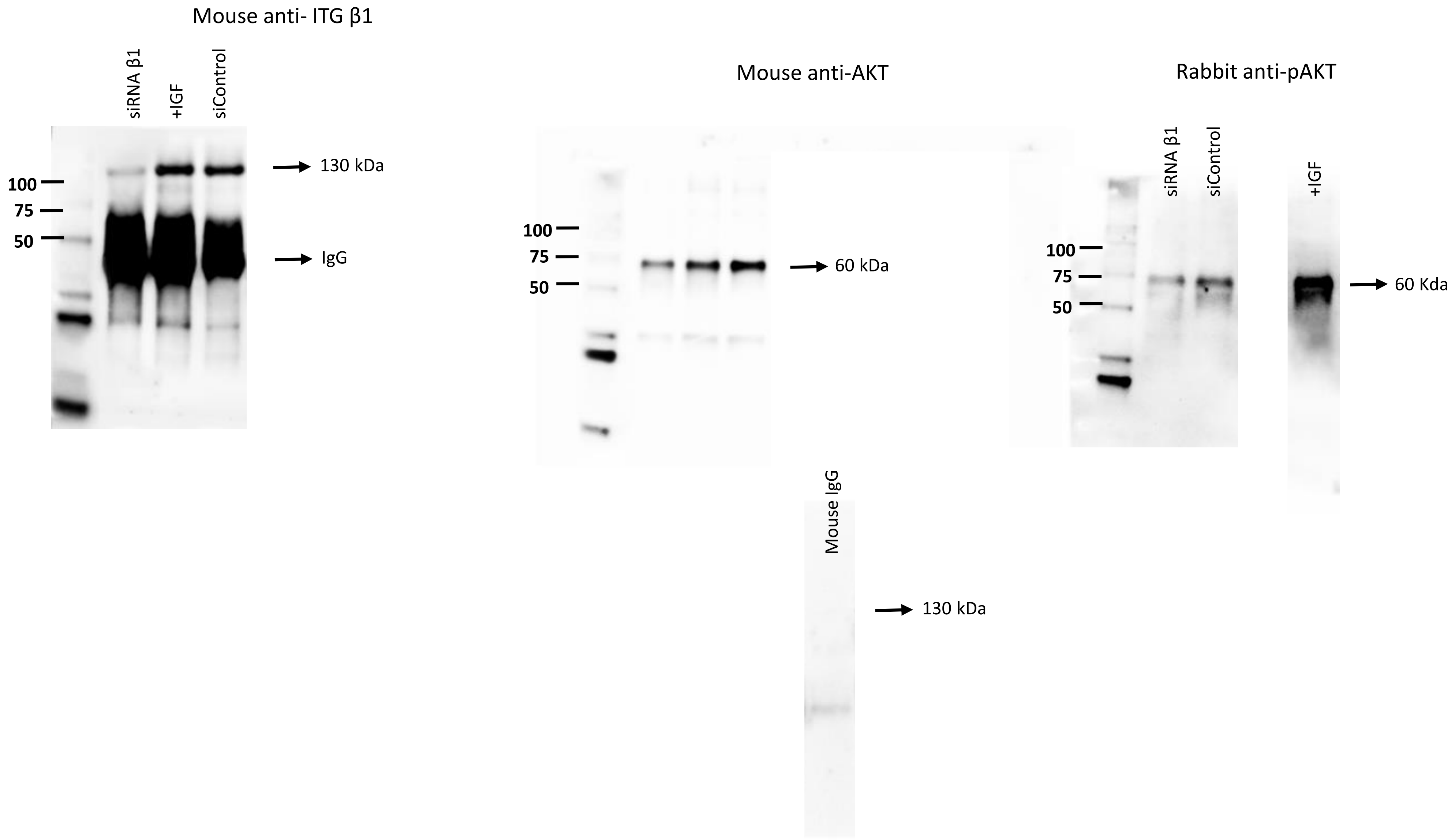

Supplement: Supplementary file 1 [file cancers-14-02430-s001.zip › cancers-1711480-supplementary.pdf]
